# Effect of Al and Fe Doping on the Electrochemical Behavior of Li_1.2_Ni_0.133_Mn_0.534_Co_0.133_O_2_ Li-Rich Cathode Material

**DOI:** 10.3390/ma15228225

**Published:** 2022-11-19

**Authors:** Anna Medvedeva, Elena Makhonina, Lidia Pechen, Yury Politov, Aleksander Rumyantsev, Yury Koshtyal, Alexander Goloveshkin, Konstantin Maslakov, Igor Eremenko

**Affiliations:** 1 Kurnakov Institute of General and Inorganic Chemistry of the Russian Academy of Sciences, 31 Leninsky pr., 119991 Moscow, Russia; 2 Ioffe Institute of the Russian Academy of Sciences, 26 Politekhnicheskaya ul., 194021 St. Petersburg, Russia; 3 A.N. Nesmeyanov Institute of Organoelement Compounds of the Russian Academy of Sciences, 28 Vavilova ul., 119334 Moscow, Russia; 4 Department of Chemistry, Lomonosov Moscow State University, Leninskiye Gory, 1/3, 119991 Moscow, Russia

**Keywords:** Li-ion battery, cathode material, Li-rich oxide, Al doping, Fe doping

## Abstract

This article studies the doping of Li-rich cathode materials. Aluminum and iron were chosen as dopants. Li-rich cathode materials for lithium-ion batteries, which were composed of Li_1.2_Ni_0.133_Mn_0.534_Co_0.133_O_2_ with a partial replacement of cobalt (2 at %) by iron and aluminum, were synthesized. The dopants were introduced at the precursor synthesis stage by co-precipitation. The presence of Fe and Al in the composition of the synthesized samples was proved by inductively coupled plasma mass spectrometry, X-ray diffraction analysis and X-ray microanalysis. The cathode materials were tested electrochemically. The incorporation of Al and Fe into the structure of lithium-enriched materials improved the cyclability and reduced the voltage fade of the cathodes. An analysis of the electrochemical data showed that the structural changes that occur in the initial cycles are different for the doped and starting materials and affect their cycling stability. The partial cation substitution suppressed the unfavorable phase transition to lower-voltage structures and improved the electrochemical performance of the materials under study.

## 1. Introduction

In the modern world, due to the constant emergence of new types of energy consumers, energy accumulation and storage systems have become an important component of the technology used for energy production. Lithium-ion batteries (LIBs) are the most in-demand type of batteries in the field of portable equipment and electric vehicles [1]. LIBS possess the highest values in terms of stored energy and operating voltage among the various types of devices for energy storage, and due to their long lifespan, LIBs have come to occupy a niche as batteries for high-power devices [2,3]. The cathode material is the key component of a LIB as it determines its electrochemical performance and makes the largest contribution to the cost of the battery [4,5]. Thus, the search for new cathode materials for further increasing the power of LIBs is very relevant [6]. Compared to traditional cathode materials with an olivine structure (LiFePO_4)_, a spinel structure (LiMn_2_O_4_) or a layered structure such as LiMO_2_ (M-3d, transition metals (TM) such as Mn, Ni, Co), lithium-rich layered oxides xLi_2_MnO_3_*(1−x)LiMO_2_ (0 < x < 1) are more promising cathode materials that are capable of providing a specific discharge capacity of about 250 mAh/g at the voltage value of about 3.5 V [7,8]. The main feature of Li-rich materials (LR-materials) is the complexity of interpreting their phase composition.

To date, researchers have not found consensus on the microstructure of LR-materials. These materials can refer to solid solutions [9,10,11,12,13], composites/nanocomposites [14,15,16,17], or integrated nanodomains [9,11]. In this study, the composition of the investigated cathode materials is described by the general formula, Li_1+y_(Ni_a_Co_b_Mn_c_)_1−y_O_2_, but it can also be written as a mixture of two phases, (1−x)Li_2_MnO_3_-xLiMO_2_ (M = Co, Ni, Mn). The latter notation emphasizes the existence of two phases in the LR-material structure: the rhombohedral LiMO_2_ (sp. gr. *R*$\bar{3}$*m*) and monoclinic Li_2_MnO_3_ (sp. gr. *C*2/*m*) phases.

The layered structure of lithium manganite (Li_2_MnO_3_) is similar to the structure of LiMO_2,_ containing TM (Ni, Co, Mn) layers alternating with lithium layers surrounded by oxygen atoms and forming honeycomb structures. However, unlike the LiMO_2_ phase, excess lithium ions in the Li_2_MnO_3_ phase occupy one third of the TM layer positions, leading to the formation of a superstructure. The structure of Li-rich materials (Li_1+y_(Ni_a_Co_b_Mn_c_)_1−y_O_2_) is similar to that of LiMO_2_, with lithium occupying some TM ion positions, in addition to octahedral sites. This is the feature that the LR-materials’ structure and the Li_2_MnO_3_ structure have in common.

LR-materials are promising as LIB cathodes due to their high discharge capacity, achieved through the electrochemical activation of the Li_2_MnO_3_ component in the first charge cycles up to voltages above 4.5 V.

Despite these advantages, there are a number of drawbacks of LR-materials [18]. These are, firstly, the low Coulombic efficiency of the first cycle caused by the irreversible release of lithium and oxygen from the material during structure transformation upon activation [19]; secondly, the low electrical conductivity of the structures; and third, the formation of a surface film on the cathode as a result of the side reactions of the electrolyte and TM [20] leads to an increase in the cell resistance. In addition, the gradual degradation in the cycling performance as a consequence of the structural evolution from a layered structure to a spinel structure at voltages above 4.5 V has been observed [21]. All these processes lead to a drop in the capacity and voltage with cycling, which is currently hampering the industrial production of LR-materials [22,23].

One of the approaches used to improve the electrochemical properties of cathode materials is a partial replacement of TM or lithium ions (doping) [24] by ions of various elements, such as Zr [25,26], Ti [27,28,29], Al [30,31], Cr [32], Fe [33,34], etc. For example, the incorporation of Mg^2+^ [35,36] and Al^3+^ [37] as dopants into LR-materials reduces cation mixing (i.e., it reduces TM migration), thereby preventing the transition of the layered structure to the spinel one. This leads to a decrease in the capacity fade and voltage decay during long-term cycling. In our previous work [36], it was shown for the first time that the incorporation of Mg^2+^ cations into TM or lithium positions has a different effect on the electrochemical characteristics. In the former case, the cyclability of the cathode material is improved even at high discharge currents. As for the latter case, the discharge capacity of the cathode is significantly reduced without any deterioration in the cycling stability and rate capabilities. The authors of [29] found that the incorporation of Ti^4+^ ions at the TM positions in the oxide cathode material significantly increases its energy capacity (285 mAh/g at a current density of 20 mA/g) and improved the cyclability of the material.

We have previously investigated the effect of cadmium modification of an LR-material composed of Li_1.2_Ni_0.133_Mn_0.534_Co_0.133_O_2_. As shown in [38], cadmium does not replace TM as it has a much larger ionic radius than that of TM (Cd^2+^—0.95 Å), but it forms a protective oxide coating on the surface of the material. The ionic radii of Fe^3+^ (0.64 Å) and Al^3+^ (0.535 Å) are close to the ionic radii of TM: Ni^2+^ (0.69 Å), Co^3+^ (0.55 Å), Mn^4+^ (0.53 Å), meaning that these cations can be introduced into the structure of LR-materials.

The doping of Li-rich cathode materials of different composition results in improved electrochemical behavior. The authors of [39] discussed the effect of the partial substitution of Ni, Co and Mn for aluminum in Ni-rich materials. The authors of [40] concluded that partial substitution for aluminum effectively suppressed the phase transformations in layered cathode materials. Sun et al. [41] found that Al doping inhibited the formation of a rock salt structure in the Li(Ni_0.61_Co_0.12_Mn_0.27_)O_2_ material, resulting in a decrease in microcrack formation. The effect of Fe doping was studied in [33]. According to the authors, doping Li-rich materials with Fe reduced the voltage decay with cycling. The doped material, Li_1.2_Mn_0.56_Ni_0.16_Co_0.03_Fe_0.05_O_2,_ described in [42] was characterized by improved cycling performance at high rates in comparison with undoped materials.

Thus, in this work we compared the effect of the partial replacement of cobalt (2 at %) with iron and aluminum in the Li_1.2_Ni_0.133_Mn_0.534_Co_0.133_O_2_ material. The doped materials demonstrated an improvement in the electrochemical performance compared with the starting material, namely, a slower decrease in the capacity and the average discharge voltage of the cathode. The nature of the dopant (Fe or Al) affects the electrochemical properties of the LR-material differently.

## 2. Materials and Methods

### 2.1. Synthesis

The following reagents were used to synthesize LR-materials: lithium hydroxide monohydrate (99%, Sigma Aldrich), manganese(II) nitrate tetrahydrate (98%, Acros Organics, Geel, Belgium), nickel(II) nitrate hexahydrate (99%, Acros Organics), cobalt(II) nitrate hexahydrate (99%, Acros Organics), aluminum(III) nitrate nonahydrate (98%, LABTECH, Moscow, Russia), ammonium iron(II) sulfate hexahydrate (99%, NORMAPUR), aqueous ammonia solution (25%, Khimmed, Moscow, Russia), and ethanol (abs., A.C.S., Merck, Rowe, NJ, USA).

The starting material, Li_1.2_Ni_0.133_Mn_0.534_Co_0.133_O_2_ (LR), and materials doped with iron Li_1.2_Ni_0.133_Mn_0.534_Co_0.118_Fe_0.016_O_2_ (LR-Fe) and aluminum Li_1.2_Ni_0.133_Mn_0.534_Co_0.118_Al_0.016_O_2_ (LR-Al) were synthesized by co-precipitation. The dopants (Fe and Al) were incorporated at the precursor synthesis stage, replacing 2 at % of cobalt in the syntheses of LR-Fe and LR-Al, respectively. 

A general procedure for the synthesis of LR-materials, exemplified by the LR-Fe material synthesis, is given below. The precursor was synthesized by co-precipitation as follows. Ni(NO_3_)_2_*6H_2_O (9.68 g), Mn(NO_3_)_2_*4H_2_O (34.19 g), Co(NO_3_)_2_*H_2_O (8.67 g) and FeSO_4_·(NH_4_)_2_SO_4_·6H_2_O (1.58 g) were dissolved in 100 mL of deionized water (DIW). To prepare a solution of the precipitant, LiOH*H_2_O (21.19 g) was dissolved in 200 mL of DIW, followed by the addition of aqueous ammonia solution (7 mL). The resulting mixture was transferred to a 250 mL volumetric flask and made up to the mark with DIW. The precipitant solution was added to a 1000 mL beaker containing 200 mL of DIW and stirred at 60 °C to adjust the pH value at 11. The aqueous mixture was constantly purged by argon flow. The solution of metal salts was added to the precipitant solution with a flow rate of 1.5 mL per min. The precipitate that formed was stored for 15 h. The precipitate was washed with DIW and dried in air at 120 °C for 20 h. The obtained precursor was carefully ground in an agate mortar with the appropriate amount of LiOH*H_2_O (excess 3 wt.% due to lithium losses during annealing) in ethanol. The resulting powder was annealed in two stages, at 480 °C for 6 h and at 900 °C for 12 h, with the intermediate homogenization of the material. The powder was carefully ground after annealing as well. The LR and LR-Al samples were synthesized in a similar manner.

### 2.2. Methods

The morphology, microstructure and uniformity of the elemental composition distribution over the obtained oxides were studied using scanning electron microscopy (SEM) and energy dispersive X-ray spectroscopy (EDX) on a NVision-40 instrument (Carl Zeiss). The particle size distribution was measured using an Analysette 22 MicroTec Plus laser analyzer.

Inductively coupled plasma optical emission spectroscopy (ICP-OES) was performed on an Thermo Scientific iCAP XP (Thermo Fisher Scientific) to determine the content of the metals in the obtained samples.

X-ray diffraction analysis (XRD) was performed at room temperature on a Bruker D8 Advance X-ray diffractometer (CuKα radiation, Ni filter), λ = 0.15418 nm, 40 kW/40 mA, Bragg-Brentano geometry) and D8 Advance X-ray Vario (CuKα1 radiation, Ge monochromator), λ = 0.1.5406 nm, 40 kW/40 mA) diffractometers for the 2θ range of 10°–90° with a step of 0.02°. The diffraction measurements of LR-Fe were carried out with a Ge monochromator; the diffraction patterns of LR and LR-Al oxides were measured without a monochromator using a Si standard reference to improve the accuracy of the cell parameters.

The X-ray photoelectron spectroscopy (XPS) measurements were performed on an Axis Ultra DLD spectrometer (Kratos Analytical, UK) using a monochromatic Al*K*α source (1486.7 eV). The pass energies of the analyzer were 160 eV for survey spectra and 40 eV for high resolution scans. The spectra were charge referenced to the lattice oxygen component in the O1s spectrum set to 529.5 eV.

The electrochemical activity of the obtained cathode materials was studied in CR2032-type coin cells on a battery tester Neware CT4008W-5V10mA. The electrode layer consisted of the active cathode material (92 wt.%), electrically conductive additive (Super C65 carbon black (Timcal)–5 wt.%) and a binder (polyvinylidene fluoride Solef 5130 (Solvay)–3 wt.%). Lithium foil was used as a negative electrode and TC-E918 (Tinci) was used as an electrolyte. After assembling the coin cells, formation cycles were conducted at a current density of 20 mA/g: two charge/discharge cycles in the voltage range of 2.5–4.3 V, followed by electrochemical activation of the material in the voltage range of 2.5–4.5 V (2 cycles), 2.5–4.6 V (2 cycles) and 2.5–4.7 V (2 cycles). Galvanostatic charge/discharge was performed at current densities ranging from 20 to 80 mA/g. The performance of the materials was also evaluated at high discharge currents in the range of 80–480 mA/g, with the charge current value remaining constant at 80 mA/g.

## 3. Results and Discussion

**Figure 1:**
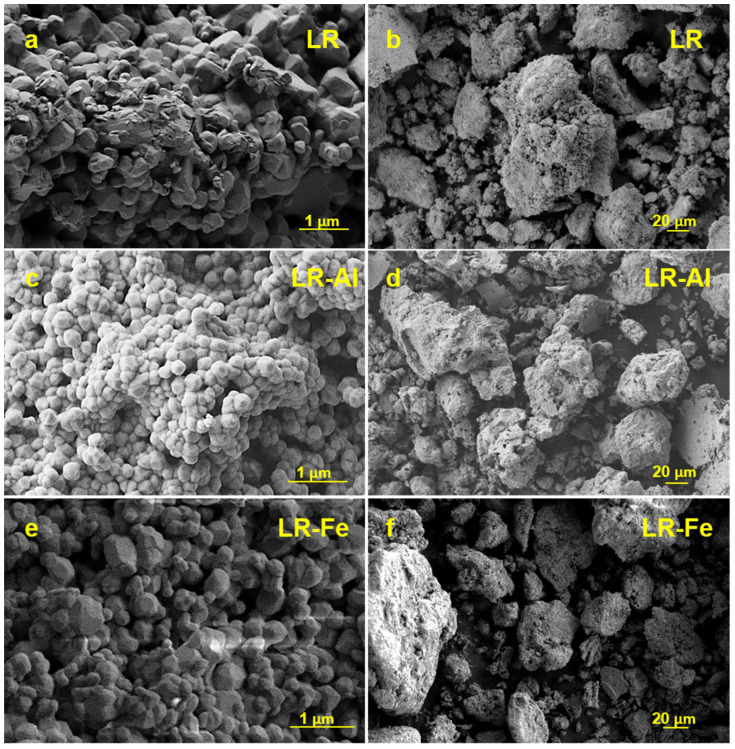
SEM images of the (**a**,**b**) LR; (**c**,**d**) LR-Al; and (**e**,**f**) LR-Fe materials.

The cathode materials synthesized from hydroxide precursors are shapeless agglomerates consisting of primary particles that are about 200 nm in size (Figure 1). A similar morphology is also characteristic of the hydroxide precursors themselves (Figure S1). According to the granulometric analysis (Figure S2), the smallest agglomerates are observed in the LR-Al material (d90 = 59.8 μm), which is characterized by a wide unimodal distribution with a small shoulder in the region of 2–10 μm (Figure S2a,b). The LR-Fe material has the largest agglomerates (d90 > 100 µm). For the starting LR sample, the distribution exhibits a bimodal pattern with a predominance of larger 20–100 µm particles (d90 = 80.1 µm). Additional grinding is required for materials consisting of agglomerates with the highest d90 parameter value (above 100 µm-sample LR-Fe) before making the electrodes.

The assumed concentrations of all elements, including Fe and Al, were confirmed by elemental analysis (Table 1). The uniform distribution of embedded elements was also proved by energy dispersive X-ray spectroscopy (Figures S3 and S4).

**Figure 2:**
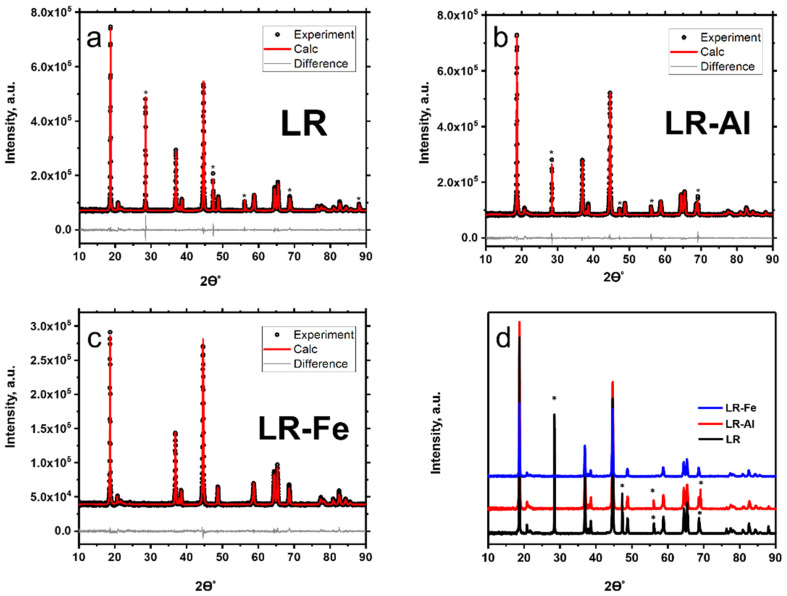
XRD patterns with Rietveld refinement for cathode materials: (**a**) LR; (**b**) LR-Al; and (**c**) LR-Fe. (**d**) overlay of diffractograms; the Si lines are designated by asterisks.

The X-ray diffraction patterns for the LR-materials demonstrated low-intensity superlattice peaks in the 2θ region between 20 and 25° (Figure 2), indicating that this phase can be indexed in the monoclinic *C*2/*m* space group, similar to the cationic ordering of the Li_2_MnO_3_ monoclinic phase [43]. The phase compositions of these materials likely refer to solid solutions based on the averaged disordered monoclinic phase Li_2_MnO_3_, due to the existence of the compatibility between the close-packed layers of two components (Li_2_MnO_3_ and LiMO_2_), with similar interlayer distances of about 4.7 Å [20] between the (001) planes in the monoclinic structure and (003) planes in the layered structure.

There are no impurity phase peaks in the diffraction patterns. The positions of the main peaks in the diffraction patterns are slightly shifted compared to the undoped sample, obviously due to the similar values of the ionic radii of the dopants and the cobalt being replaced. At the same time, the change in the lattice parameters with the incorporation of dopants is significant (Table 2), which confirms the substitution of cobalt by aluminum or iron.

The survey XPS spectra of the samples (Figure S5) show lines of manganese, nickel, cobalt, oxygen, and carbon. Table S1 summarizes the concentrations of the elements on the surface of the samples, calculated from the high-resolution XPS spectra.

**Figure 3:**
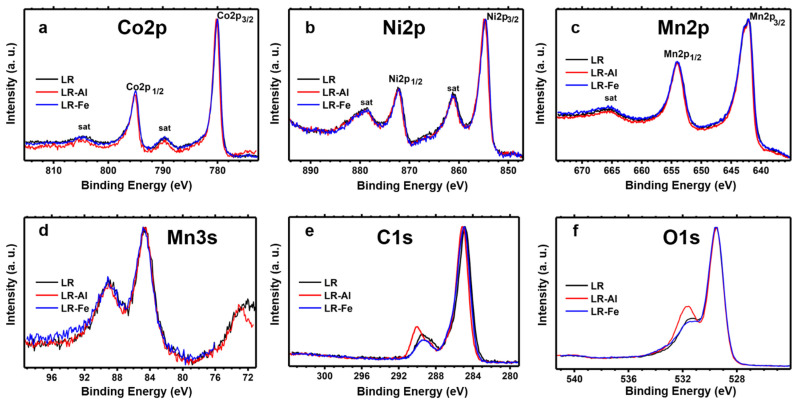
Normilized high-resolution (**a**) Co2p, (**b**) Ni2p, (**c**) Mn2p, (**d**) Mn3s, (**e**) C1s, and (**f**) O1s XPS spectra of LR, LR-Al and LR-Fe.

The high-resolution spectra are shown in Figure 3. The Co2p spectra (Figure 3a, Table S2) correspond to the trivalent state of cobalt. Intense satellites observed in the Ni2p spectra of the samples (Figure 3b) are typical for divalent nickel compounds. The Mn2p and Mn3s spectra (Figure 3c,d) are more characteristic of tetravalent manganese and they are close to the spectra of MnO_2_ manganese oxide (Table S3). The spectra of manganese, nickel, and cobalt lines practically coincide for all the samples. The presence of carbonates is indicated by a characteristic component in the C1s spectra at about 290 eV (Figure 3e and Figure S6). The other components in these spectra at binding energies of about 285.2, 286.8, and 288.8 eV correspond to C−C, C−O, and O=C−O species. The O1s spectra (Figure 3f and Figure S7) contain a component of lattice oxygen at a binding energy of 529.5 eV and two less intense components at higher binding energies. The components at about 531.4 and 533.5 eV can be assigned respectively to lithium carbonates and oxygen bonded to adventitious carbon.

**Figure 4:**
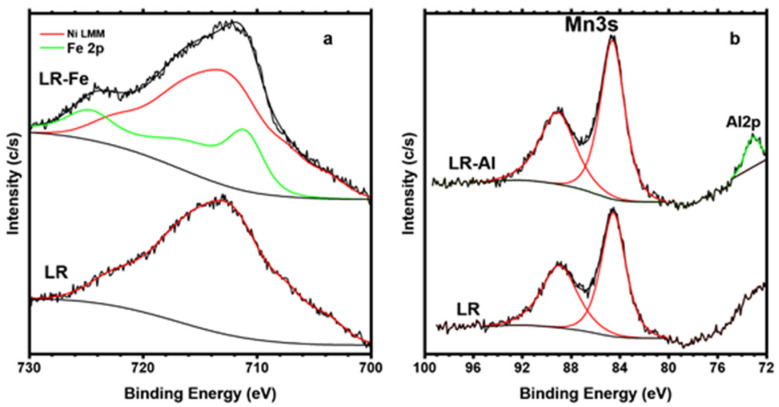
(**a**) NiLMM Auger and Fe2p XPS spectra of LR and LR-Fe and (**b**) Mn3s and Al2p XPS spectra of LR and LR-Al.

The Fe2p line of the LR-Fe sample overlaps with the more intense NiLMM Auger line (Figure 4a). However, using the spectrum of the iron-free LR sample as a reference NiLMM component, the Fe2p component can be distinguished qualitatively. The resulting position and shape of the Fe2p component in the spectrum of the LR-Fe sample indicates the trivalent state of iron. The Al2p line in the spectrum of the LR-Al sample is relatively weak and overlaps with the high energy tail of the Ni3s line (Figure 4b).

**Figure 5:**
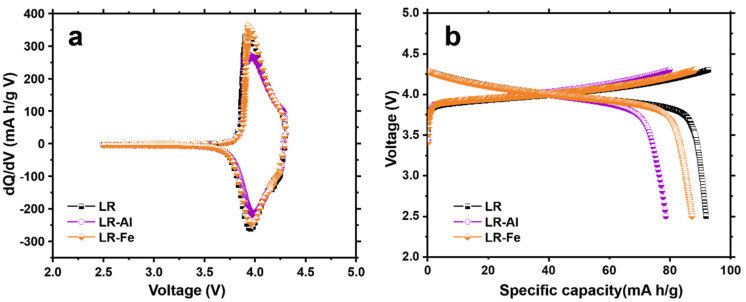
(**a**) dQ/dV plots and (**b**) the charge–discharge curves in the second cycle in the potential range of 2.5–4.3 V.

To obtain high values of a specific capacity, the complex structures of LR-materials require preliminary electrochemical activation at high voltage. For the activation/formation scheme, see the Materials and Methods section. The curves of the first derivative of capacity with respect to voltage (dQ/dV) vs. voltage allow us to trace the changes occurring in the material structure during cycling and to evaluate the effect of doping on these changes. In order to better understand the changes taking place, the formation process is discussed in more detail. The charge/discharge and dQ/dV curves of the second formation cycle (2.5–4.3 V) for all of the materials are shown in Figure 5. Lithium is extracted from the octahedral positions of the LR-material structure in the voltage range of 2.5–4.3 V. The anodic curve peaks are responsible for the oxidation of TM, and the cathode curve peaks are responsible for their reduction (Figure 5a), with all three materials showing similar patterns [43]. One can see from the charge/discharge curves (Figure 5b) that the maximum discharge capacity value is achieved for the LR sample (91.9 mAh/g), while the corresponding values for the doped materials are lower: LR-Fe (87.2 mAh/g) and LR-Al (78.7 mAh/g). Note that, according to a number of authors [33,34,44], iron, as a transition d-element (in contrast to the non-transition p-element Al), can also participate in the electrochemical reaction in the initial cycles (at voltage above 4.0 V) and contribute to the LR-material capacity. Indeed, when replacing the same amount of Co in the LR-material with Fe and Al, LR-Fe demonstrates higher charge and discharge capacities than LR-Al. According to [34,45], the Fe^4+^ ion is unstable and forms an electrochemically inactive LiFeO_2_ phase.

**Figure 6:**
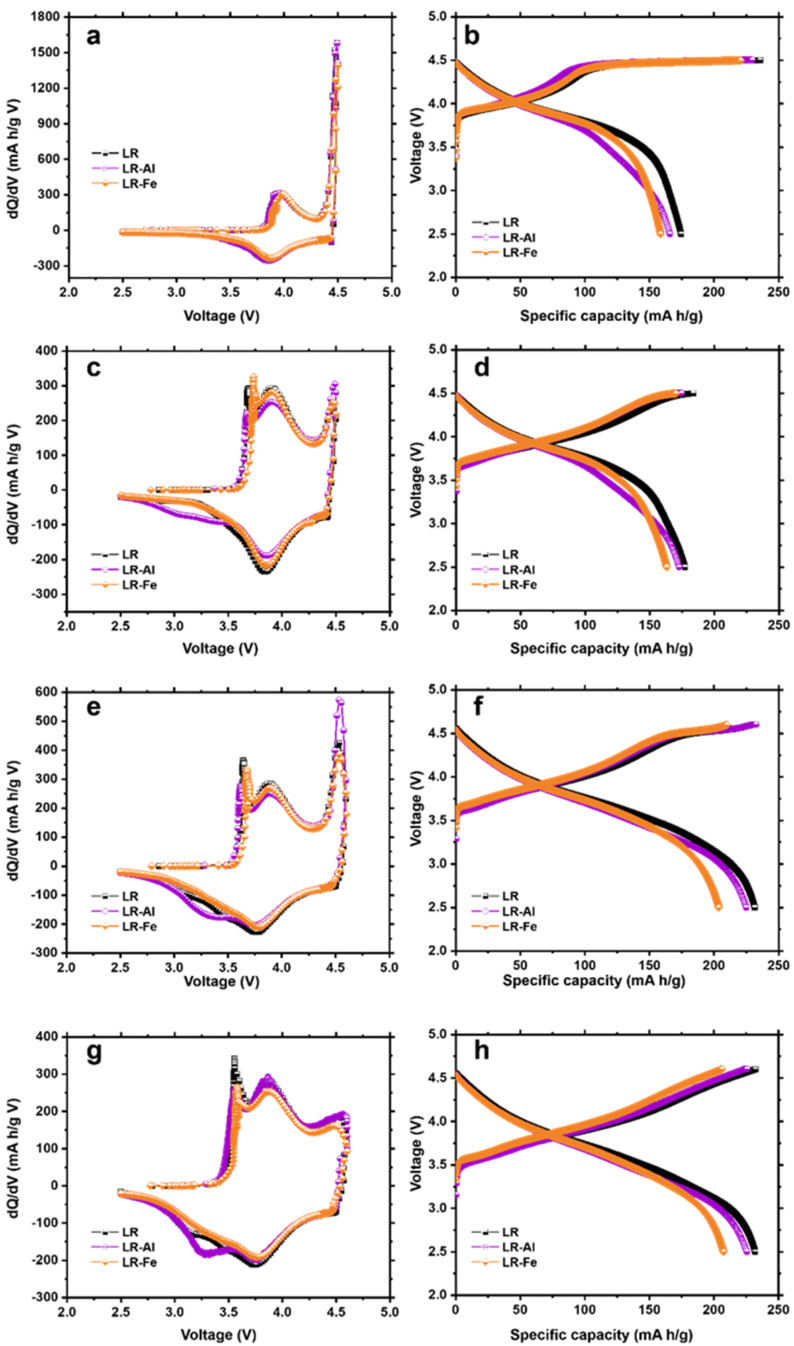
(**a**,**c**,**e**,**g**) dQ/dV plots and (**b**,**d**,**f**,**h**) corresponding charge–discharge curves for the 3–6 cycles in the potential ranges of (**a**–**d**) 2.5–4.5 V and (**e**–**h**) 2.5–4.6 V.

Upon increasing the charge voltage above 4.5 V, the material activation effect appears. Lithium is extracted from the TM positions (formation cycles 3 and 4, Figure 6a–d), which is reflected in the dQ/dV curve by the appearance of a sharp peak at the given voltage (Figure 6a,c).

According to the literature data [43], this peak is attributed to the oxidation of oxygen, which indicates the beginning of the material structure transformation. The complex process of extracting a Li^+^ ion from electrodes based on LR-materials at voltage above 4.5 V includes the simultaneous oxidation of oxygen (with its possible release) and the rearrangement of TM ions. This process can be described by the following equation:

Li_2_MnO_3_→2Li^+^ +2e^−^ + MnO_2_ + 1/2O_2_↑ (1)

Modern studies [46,47] using online electrochemical mass spectrometry (OEMC) and high-resolution transmission microscopy (HR-TEM) indicate that the irreversible extraction of oxygen occurs from the LR-material near-surface layer a few nm thick, rather than from the volume of Li_2_MnO_3_ monoclinic phase particles. Note that in addition to the release of gaseous oxygen [14,48] when charged to high voltages, oxygen can be reversibly oxidized to peroxo-groups (2O^2−^ ↔ O_2_ ^2−^ + e^−^). This reversible transition from oxo-(O^2−^) to peroxo-group (O_2_)n-occurring during the charge/discharge process of LR-materials is driven by the reductive coupling mechanism described in [49,50]. The shape of the charge profiles for the third cycle (Figure 6b) differs from that for the second cycle. These curves have an inclined region (3.7 V–4.4 V) and a plateau region (4.4 V–4.7 V). The inclined region, also present in the second cycle charge curves, reflects the course of the TM oxidation process. LR-Fe demonstrates a larger capacity contribution at this stage than LR-Al and almost coincides with LR. The plateau region corresponds to the oxidation process of O^2−^ ions and TM ions above the oxidation state 4^+^ [44]. The increase in charge capacity due to oxygen oxidation, taking place during the monoclinic structure rearrangement, is maximum for undoped LR. The evolution of the LR-materials structure during cycling suggests a gradual transition to lower-voltage spinel-like structures [51].

The rhombohedral structure is another structure related to LR-materials. Layered Li-MO_2_ also gradually transforms to the spinel structure [15]. This transition starts from the particles surface and spreads to the entire material volume during cycling as a result of gradual migration of TM to lithium positions and displacement of lithium ions to tetrahedral positions.

A large irreversible capacity is typical for Li-rich materials in the first cycles with an increase in the cycling voltage above 4.5 V. This is caused by the ongoing transformation of the cathode material structure. As lithium ions are extracted from the material first from the Li layer and then from the layers of transition metals, further oxidation of oxygen ions occurs. This process is partially irreversible because the atomic oxygen formed can transform into molecular oxygen and lost from the material, or participate in side reactions with the electrolyte. Additionally, some of the lithium ions are spent on the formation of electrode films. All this leads to a large loss of capacity in the first cycles.

The gradual completion of the monoclinic structure rearrangement (oxygen peak disappearance) occurs at formation cycle 4–6 (Figure 6c–h). Starting from cycle 4, the anodic curve exhibits peaks in the voltage range of 3.7 V, related to the oxidation of Mn^3+^. On the cathodic curve, a peak correlated with the reduction of Mn^4+^ to Mn^3+^ gradually increases in the region of 3.3–3.4 V [51,52]. Upon increasing the voltage in cycle 5 to 4.6 V (Figure 6f), the plateau is much shorter than in cycle 3, and it disappears in cycle 6.

**Figure 7:**
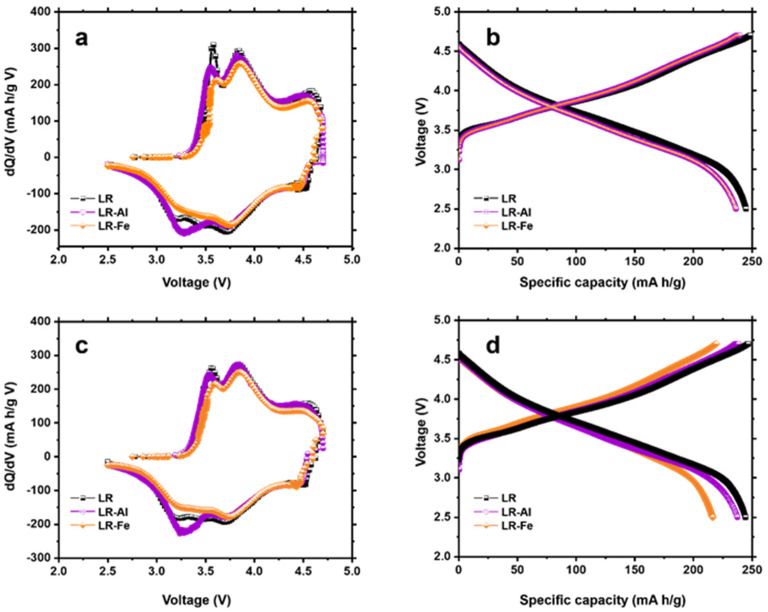
(**a**,**c**) dQ/dV) plots and (**b**,**d**) corresponding charge–discharge curves for the 7, 8 cycles in the potential ranges of 2.5–4.7 V.

Cycles 7 and 8 (Figure 7) complete the activation process. By the last activation cycle (Figure 7c), the shapes of the dQ/dV curves are similar for all the materials obtained. Characteristic peaks on the cathode curve at ~4.3, ~3.8, and 3.3 V are attributed to the reduction reactions of O, Ni/Co, and Mn, respectively [52]. The Mn reduction peak is least pronounced for the LR-Fe sample. The maximum discharge capacity (Figure 7d) was obtained for the LR sample (244 mAh/g), and a very close value was achieved for LR-Al (238 mAh/g). The discharge capacity value for LR-Fe is lower than that for the starting sample by more than 10% (216 mAh/g). Capacity reversibility (Coulombic efficiency) is also better for an undoped sample.

**Figure 8:**
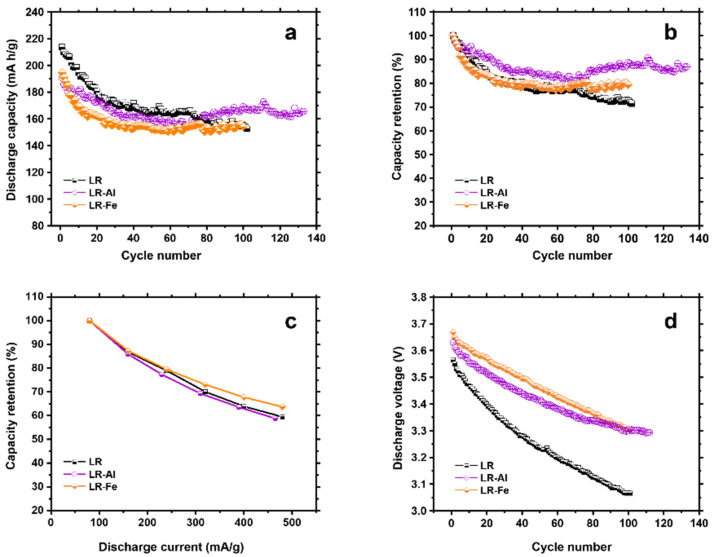
Electrochemical performance of LR, LR-Al, and LR-Fe: (**a**) the discharge capacity as a function of cycle number at the current densities of 80 mA/g; (**b**) the capacity retention vs. cycle number at 80 mA/g; (**c**) rate performance; and (**d**) average discharge voltage fade as a function of cycle number.

The results of electrochemical testing of the samples after activation are shown in Figure 8. The discharge capacity (Figure 8a,b) is initially maximum for LR, but the capacity fade during cycling is greater for this sample than for the doped LR-Al and LR-Fe materials. At cycle 80, the capacity values are equal for all samples, while for LR-Al they subsequently begin to exceed the LR values. As noted above, the capacity fade and voltage decay in LR-materials are associated with structural phase transformations. This is well illustrated in Figure 8b, which shows the capacity retention of the samples as a percentage of the first cycle. The discharge profile of the LR-Al material is flatter than that for the LR sample, and the capacity loss is minimal for the former. Starting from cycle 40, the capacity values experience almost no change for LR-Al, indicating the stabilization of the working material structure. During long cycling, the capacity values for LR-Fe appear to exceed those for the undoped LR. For the undoped sample, the discharge capacity after 100 cycles was 73% of the initial value, while for the materials doped with iron and aluminum, it was 79% and 89% of the initial capacity, respectively.

When cycling at increased current densities, the LR-Fe sample showed slightly better results (Figure 8c). However, undoped LR also demonstrated high values in terms of the discharge capacity absolute value. This is due, firstly, to the fact that the dopants (iron and aluminum) partially replace electrochemically active cobalt. Secondly, dopants do not increase the ionic and electronic conductivity, hence the performance is low when operating at high currents. The incorporation of dopants also slowed down the discharge voltage drop during the charge/discharge cycling of the modified materials (Figure 8d). For both doped samples, the average discharge voltage after 100 cycles was 3.3 V, while for the starting sample it was equal to 3.07 V. This is indicative of the suppression of phase transitions from layered and monoclinic structures to lower-voltage spinels.

**Figure 9:**
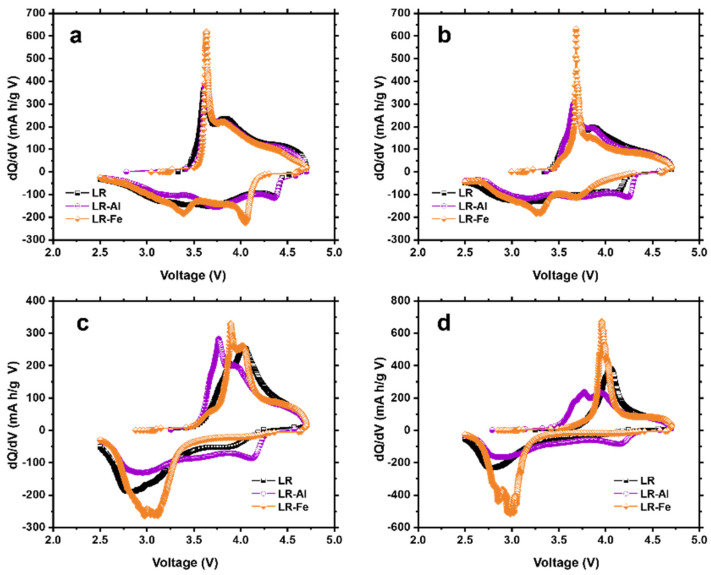
dQ/dV plots for (**a**) 2, (**b**) 25, (**c**) 75, and (**d**) 100 cycles.

Structural changes that occur in the course of cycling are reflected in the dQ/dV curves shown in Figure 9. The main change is the Mn reduction peak shift from 3.3 V towards lower voltages. The largest shift is observed for the LR sample (Figure 9d), characterized by a drop in the voltage value to 2.8 V. The shifts are smaller for doped samples: to 2.9 V for LR-Al and to 3 V for LR-Fe. These data are consistent with the voltage drop curves (Figure 8d) and confirm the assumption about the suppression of phase transformations in doped materials.

## 4. Conclusions

Lithium-rich cathode materials, Li_1.2_Ni_0.133_Mn_0.534_Co_0.133_O_2_ with a partial replacement of cobalt by iron or aluminum, were synthesized and studied electrochemically. The materials doped with Fe and Al showed lower specific discharge capacity values compared to the starting LR sample. This is appears to be associated with a decrease in the concentration of electrochemically active cobalt, as well as with the formation of LiFeO_2_ in case of LR-Fe. At the same time, both doped samples demonstrate better cyclability, and their discharge capacity values approach each other over time. In particular, for the sample doped with aluminum (LR-Al), the capacity value begins to exceed that of LR during cycling. The rate capabilities of the samples are close, with the LR-Fe sample demonstrating somewhat better results compared to the undoped material. Both samples also showed a significant decrease in the voltage drop during cycling, which indicates the suppression of ongoing phase transitions. Perhaps, the incorporation of Al and Fe promotes a decrease in the transition metal migration, thereby stabilizing the structure.

## Figures and Tables

**Table 1:** ICP-OES analysis results.

Sample	Determined Composition	Targeted Composition
LR	Li_1.2_Ni_0.133_Mn_0.534_Co_0.133_O_2_	Li_1.2_Ni_0.133_Mn_0.534_Co_0.133_O_2_
LR–Al	Li_1.2_Ni_0.133_Mn_0.534_Co_0.118_Al_0.016_O_2_	Li_1.2_Ni_0.134_Mn_0.531_Co_0.118_Al_0.017_O_2_
LR–Fe	Li_1.2_Ni_0.133_Mn_0.534_Co_0.118_Fe_0.016_O_2_	Li_1.2_Ni_0.133_Mn_0.532_Co_0.118_Fe_0.018_O_2_

**Table 2:** Refined unit cell parameters of LR, LR-Al, and LR-Fe materials (space group *C*2/*m*).

Sample	*a*, Å	*b*, Å	*c*, Å	*β*, °	*V*, Å^3^
LR	4.93909(6)	8.55501(11)	5.02970(11)	109.259(3)	200.632(8)
LR-Al	4.93922(6)	8.55523(10)	5.02635(13)	109.283(3)	200.478(8)
LR-Fe	4.9441(4)	8.5638(7)	5.0321(5)	109.286(2)	201.10(4)

## Data Availability

The data presented in this study are available on request from the corresponding authors.

## Supplementary Materials

The following are available online at https://www.mdpi.com/article/10.3390/ma15228225/s1, Figure S1: SEM micrographs of samples (a,b) R, (c,d) R–Al, (e,f) R–Fe, Figure S2: Differential and integral size distribution curves of agglomerates of the synthesized samples, Figure S3: (a) SEM micrograph of LR–Al sample and (b–f) element distribution maps: (b) Al, (c) Mn, (d) Ni, (e) O, and (f) Co, Figure S4: (a) SEM micrograph of LR–Fe sample and (b–f) element distribution maps: (b) Fe, (c) Mn, (d) Ni, (e) O, and (f) Co., Figure S5. Survey XPS spectra of LR, LR-Al and LR-Fe samples., Figure S6. C1s spectra of LR, LR-Al and LR-Fe samples, Figure S7. O1s spectra of LR, LR-Al and LR-Fe, Table S1. Concentrations of elements on the surface of the studied samples, calculated from high-resolution XPS spectra, Table S2. Binding energies of the Co2p_3/2_ lines and the relative position of the satellite (ΔCo2p_sat_) in the XPS spectra of the studied samples and reference cobalt compounds. Table S3. Binding energies of Mn2p3/2 and Mn3s XPS spectra, relative satellite position (ΔMn2p_sat_) in Mn2p_1/2_ XPS spectra, splitting (ΔMn3s) of Mn3s XPS spectra of the studied samples and reference manganese oxides. References [53,54,55,56,57,58,59,60] are cited in the supplementary materials.
