# Supplementary material for: Effect of Al and Fe Doping on the Electrochemical Behavior of Li1.2Ni0.133Mn0.534Co0.133O2 Li-Rich Cathode Material
Source: Materials (Basel). 2022 Nov 19;15(22):8225. doi: 10.3390/ma15228225 (PMC9697585; doi:10.3390/ma15228225)
Supplement: Supplementary file 1 [file materials-15-08225-s001.zip › materials-2012007-supplementary.pdf]

Supplementary materials

Effect of Al and Fe Doping on the Electrochemical Behavior of $Li_{1.2}Ni_{0.133}Mn_{0.534}Co_{0.133}O_2$ Li-Rich Cathode Material

Anna Medvedeva [1,*], Elena Makhonina [1], Lidia Pechen [1], Yury Politov [1], Aleksander Rumyantsev [2], Yury Koshtyal [2], Alexander Goloveshkin [3], Konstantin Maslakov [4] and Igor Eremenko [1,*]

[1] Kurnakov Institute of General and Inorganic Chemistry of the Russian Academy of Sciences, 31 Leninsky pr., 119991 Moscow, Russia
[2] Ioffe Institute of the Russian Academy of Sciences, 26 Politekhnicheskaya ul., 194021 St. Petersburg, Russia
[3] A.N. Nesmeyanov Institute of Organoelement Compounds of the Russian Academy of Sciences, 28 Vavilova ul., 119334 Moscow, Russia
[4] Department of Chemistry, Lomonosov Moscow State University, Leninskiye Gory, 1/3, 119991 Moscow, Russia
* Correspondence: anna.ev.medvedeva@gmail.com (A.M.); ilerem@igic.ras.ru (I.E.); Tel.: +7-495-952-07-87 (A.M.)

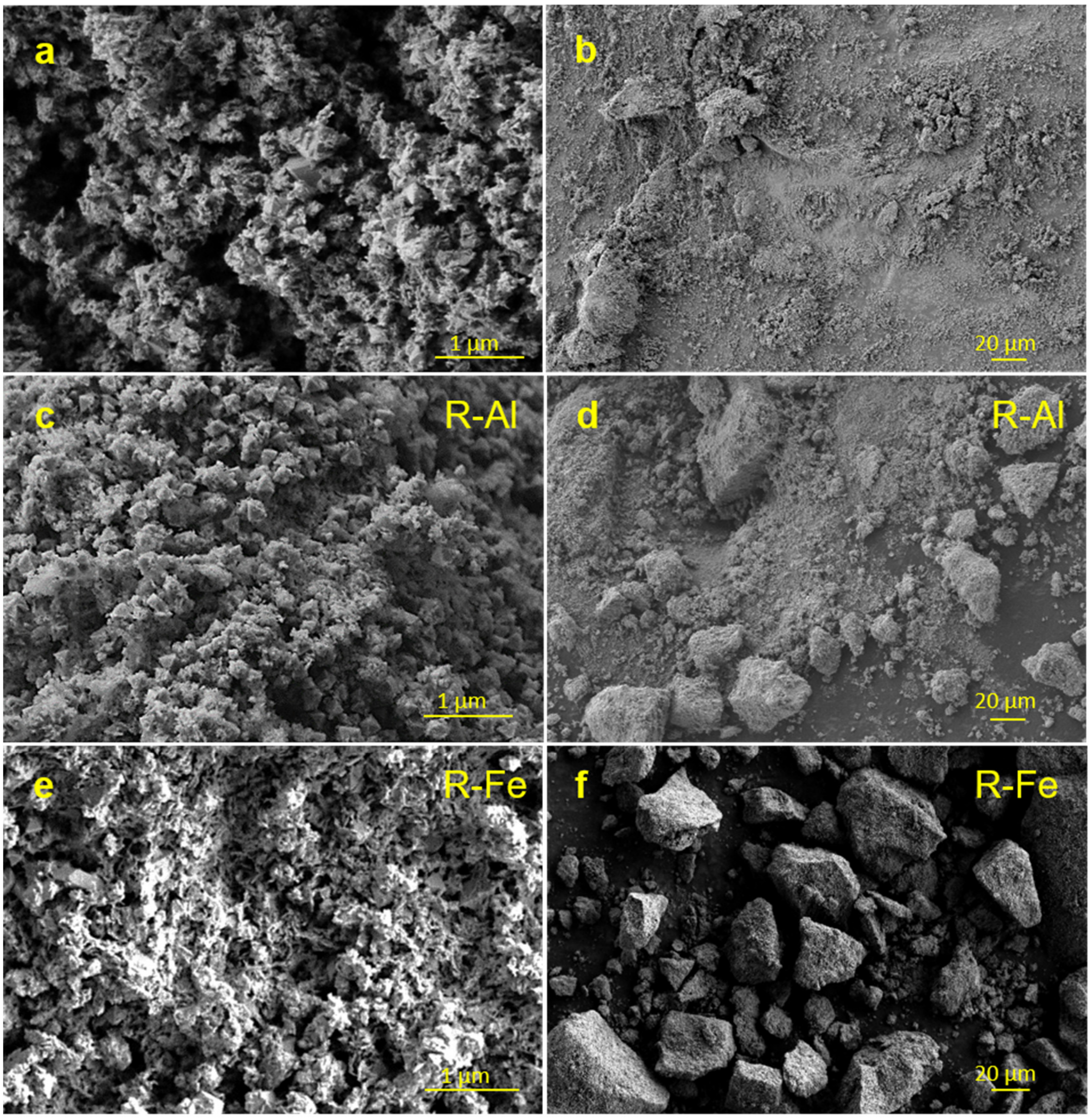

Figure S1. SEM micrographs of (a, b) R, (c, d) R – Al, and (e, f) R – Fe samples.

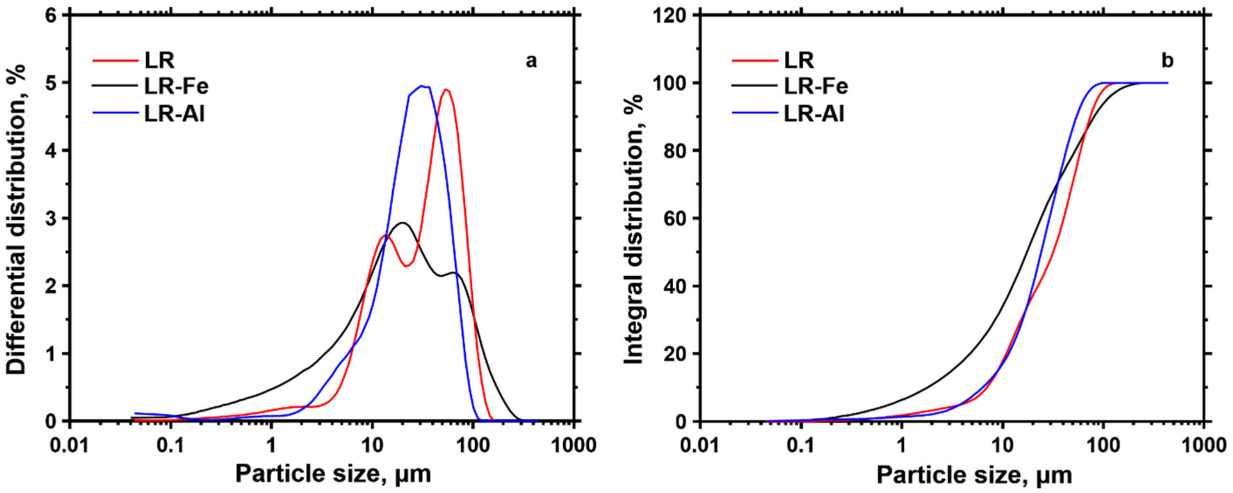

Figure S2. Differential and integral size distribution curves of agglomerates of the synthesized samples.

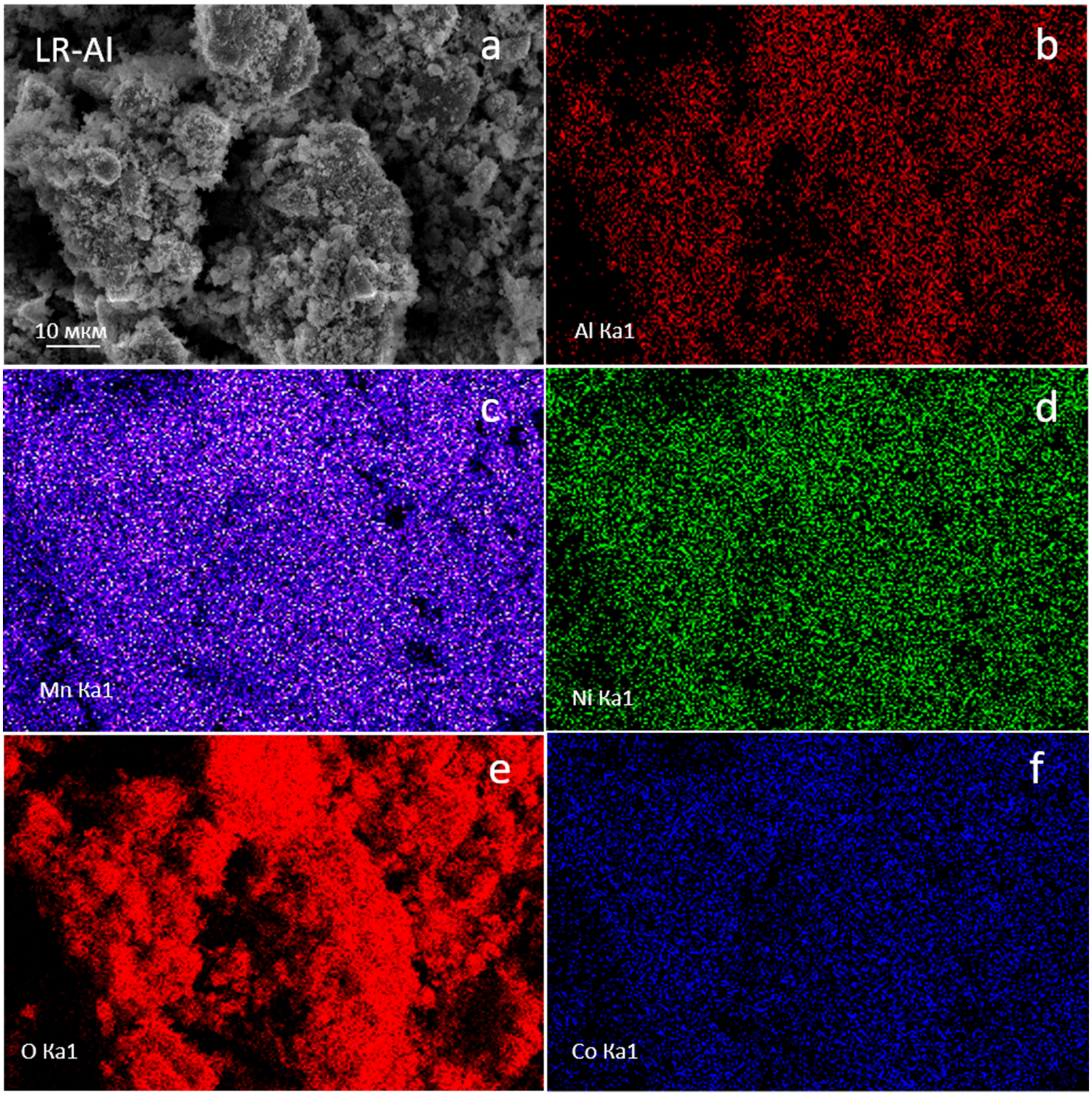

Figure S3. (a) SEM micrograph of LR – Al sample and (b-f) the element distribution maps: (b) Al, (c) Mn, (d) Ni, (e) O, and (f) Co.

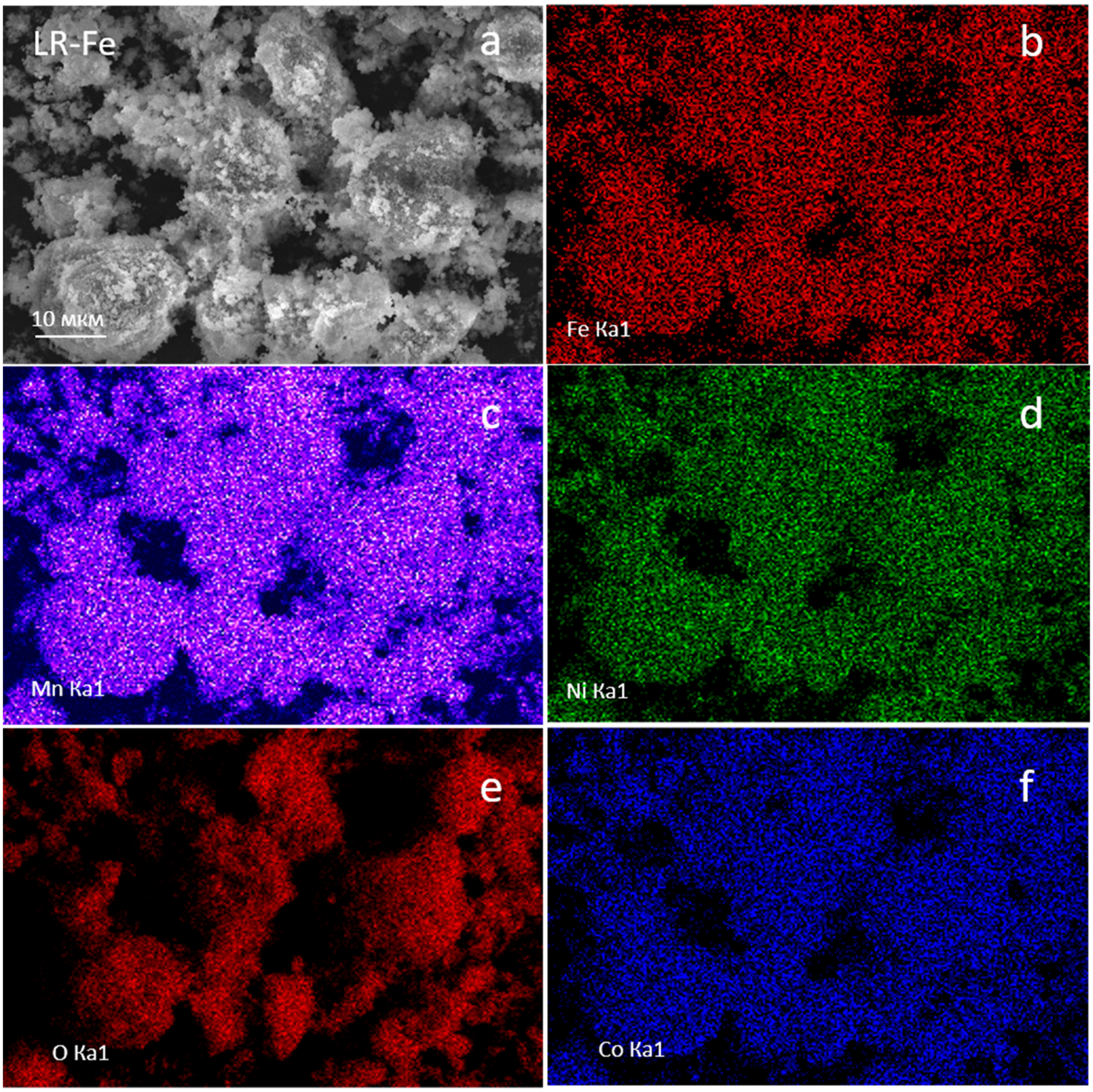

Figure S4. (a) SEM micrograph of LR – Fe sample and (b-f) element distribution maps: (b) Fe, (c) Mn, (d) Ni, (e) O, and (f) Co.

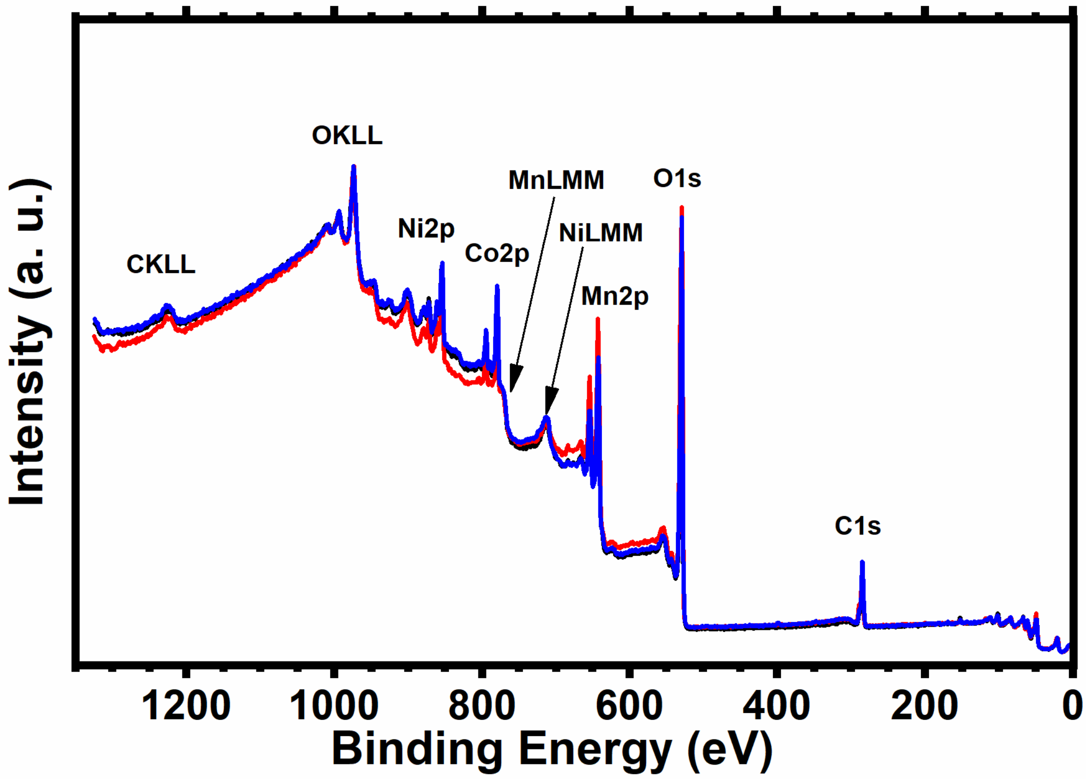

Figure S5. Survey XPS spectra of the samples LR, LR-Al and LR-Fe samples.

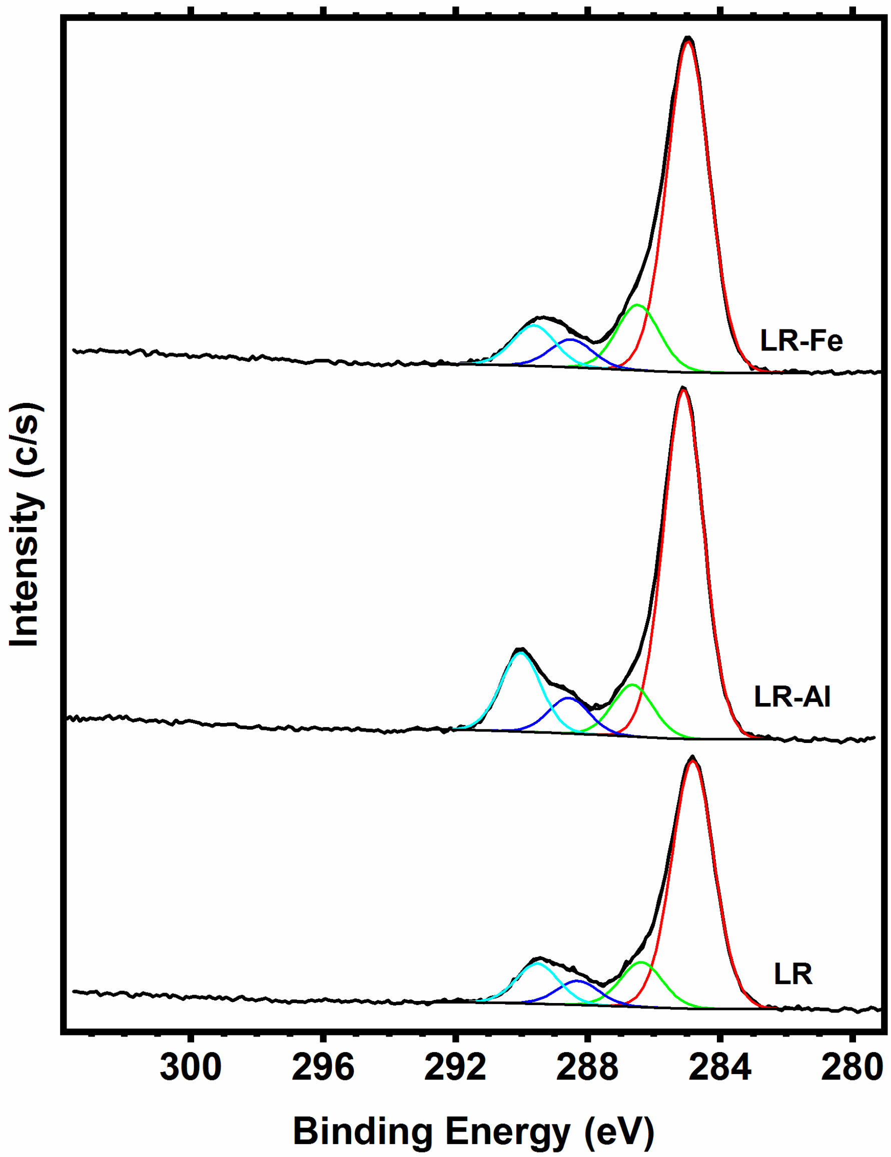

Figure S6. C1s spectra of LR, LR-Al and LR-Fe samples.

.

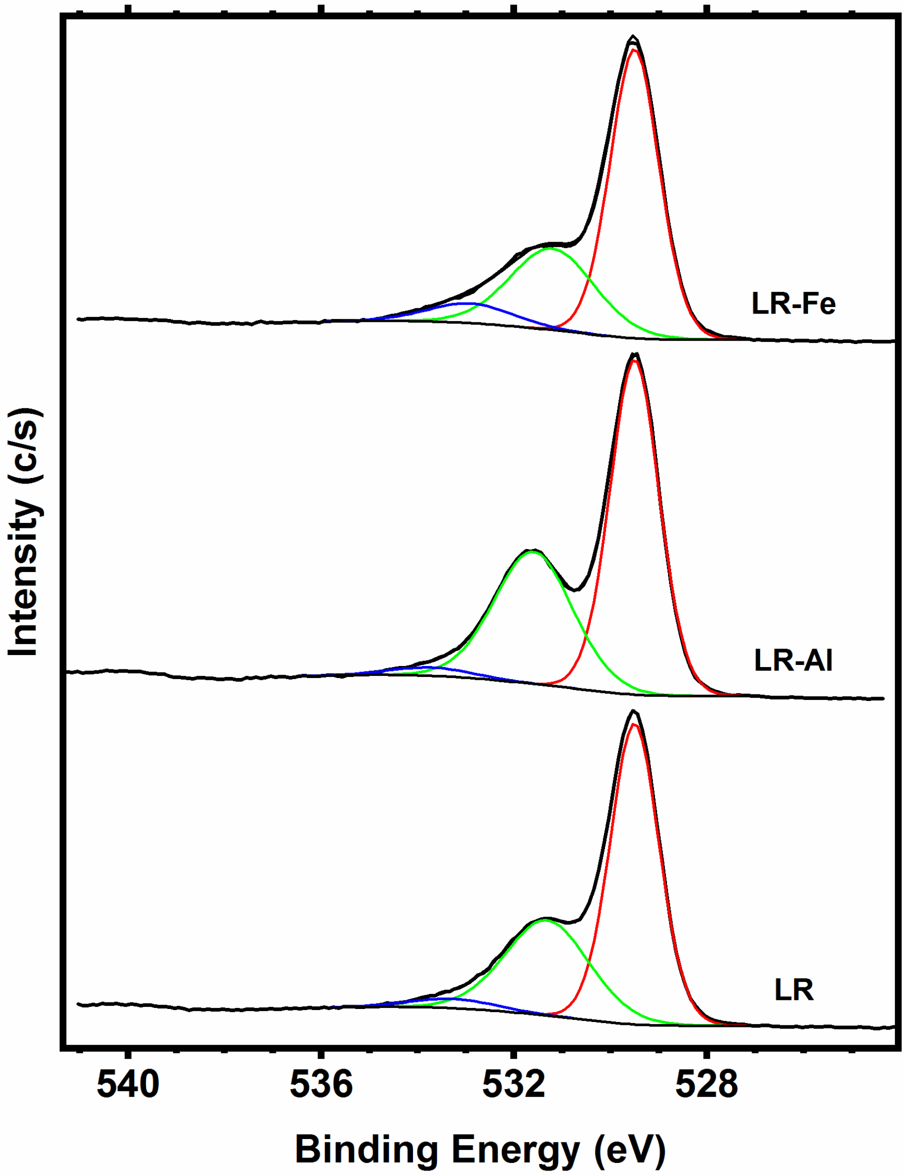

Figure S7. O1s spectra of LR, LR-Al and LR-Fe.

Table S1. Concentrations of elements on the surface of the studied samples (at %), calculated from the high-resolution XPS spectra

Samples	Ni	Co	Mn	O	C	Fe	Al
**LR**	6.2	4.9	11.8	55.6	21.5	–	–
**LR-Al**	4.7	2.9	11.8	54.9	25.1	–	0.6
**LR-Fe**	5.2	4.7	10.4	51.3	26.9	1.5	–

Table S2. Binding energies of the $Co2p_{3/2}$ lines and the relative position of the satellite ($\Delta Co2p_{sat}$) in the XPS spectra of the studied samples and reference cobalt compounds, eV.

Samples	$Co2p_{3/2}$	$\Delta Co2p_{sat}$	Ref.
**LR**	780.0	9.6	
**LR-Al**	780.2	9.6	
**LR-Fe**	780.0	9.6	
**$ScCo_{0.95}Fe_{0.95}O_3$ (Co^{3+})**	780.2	10.2	[53]
**$Co(OH)_2$ (Co^{2+})**	781.2	5.0	[54]
**$LiCoO_2$ (Co^{3+})**	780.2	9.9	[55]
**$LiNi_{0.4}Mn_{0.4}Co_{0.2}O_2$ (Co^{3+})**	780.2	9.5	[55]
**$LiCoO_2$ (Co^{3+})**	779.6	8.6	[56]
**$Li_{0.5}Co_{0.5}Ni_{0.5}O_2$ (Co^{3+})**	779.4	9.0	[56]

Table S3. Binding energies of Mn2p3/2 and Mn3s XPS spectra, relative satellite position ($\Delta Mn2p_{sat}$) in $Mn2p_{1/2}$ XPS spectra, splitting (ΔMn3s) of Mn3s XPS spectra of the studied samples and reference manganese oxides, eV.

Samples	$Mn2p_{3/2}$	$\Delta Mn2p_{sat}$	Mn3s	ΔMn3s	O1s	Ref.
**LR**	642.1	11.4	84.6	4.4	529.5	
**LR-Al**	642.1	11.4	84.6	4.5	529.5	
**LR-Fe**	642.1	11.4	84.7	4.3	529.5	
**MnO (Mn^{2+})**	640.8	~6.0	83.1	6.1	529.6	[57,58]
**Mn_2O_3 (Mn^{3+})**	641.8	10.1	83.8	5.4	530.0	[59]
**MnO_2 (Mn^{4+})**	642.4	11.8	84.7	4.4	529.6	[60]
